# Network analysis of coronary artery disease risk genes elucidates disease mechanisms and druggable targets

**DOI:** 10.1038/s41598-018-20721-6

**Published:** 2018-02-21

**Authors:** Harri Lempiäinen, Ingrid Brænne, Tom Michoel, Vinicius Tragante, Baiba Vilne, Tom R. Webb, Theodosios Kyriakou, Johannes Eichner, Lingyao Zeng, Christina Willenborg, Oscar Franzen, Arno Ruusalepp, Anuj Goel, Sander W. van der Laan, Claudia Biegert, Stephen Hamby, Husain A. Talukdar, Hassan Foroughi Asl, CVgenes@target consortium, Martin Dichgans, Tobias Dreker, Mira Graettinger, Philip Gribbon, Thorsten Kessler, Rainer Malik, Matthias Prestel, Barbara Stiller, Christine Schofield, Gerard Pasterkamp, Hugh Watkins, Nilesh J. Samani, Timo Wittenberger, Jeanette Erdmann, Heribert Schunkert, Folkert W. Asselbergs, Johan L. M. Björkegren

**Affiliations:** 1 Genedata AG, Basel, Switzerland; 2 Institute for Cardiogenetics, Lübeck, Germany; 3 Division of Genetics and Genomics, The Roslin Institute, University of Edinburgh, Edinburgh, United Kingdom; 4 Clinical Gene Networks AB, Stockholm, Sweden; 5 Department of Cardiology, Division Heart and Lungs, University Medical Center Utrecht, University of Utrecht, Utrecht, The Netherlands; 6 Deutsches Herzzentrum München, Klinik für Herz- und Kreislauferkrankungen, Technische Universität München, Munich, Germany; 7 DZHK (German Centre for Cardiovascular Research), Munich Heart Alliance, Munich, Germany; 8 Department of Cardiovascular Sciences, University of Leicester and NIHR Leicester Biomedical Research Centre, Leicester, United Kingdom; 9 Division of Cardiovascular Medicine, Radcliffe Department of Medicine, John Radcliffe Hospital, Oxford, United Kingdom; 10 Department of Genetics and Genomic Sciences, Icahn Institute for Genomics and Multiscale Biology, Icahn School of Medicine at Mount Sinai, New York, USA; 11 Laboratory of Experimental Cardiology, Department of Cardiology, Division Heart and Lungs, University Medical Center Utrecht, University of Utrecht, Utrecht, The Netherlands; 12 Integrated Cardio Metabolic Centre, Department of Medicine, Karolinska Institutet, Karolinska Universitetssjukhuset, Huddinge, Sweden; 13 Laboratory of Clinical Chemistry and Hematology, Division Laboratories and Pharmacy, University Medical Center Utrecht, University of Utrecht, Utrecht, The Netherlands; 14 Institute of Cardiovascular Science, Faculty of Population Health Sciences, University College London, London, United Kingdom; 15 Ludwig-Maximilians-Universität, Munich, Germany; 16 4SC Discovery GmbH, Mainz, Germany; 17 Fraunhofer Gesellschaft Zur Forderung Der Angewandten Forschung Ev, Munich, Germany; 18 Deutsches Herzzentrum München, Munich, Germany; 19 Horizon Discovery Limited, Cambridge, United Kingdom

## Abstract

Genome-wide association studies (GWAS) have identified over two hundred chromosomal loci that modulate risk of coronary artery disease (CAD). The genes affected by variants at these loci are largely unknown and an untapped resource to improve our understanding of CAD pathophysiology and identify potential therapeutic targets. Here, we prioritized 68 genes as the most likely causal genes at genome-wide significant loci identified by GWAS of CAD and examined their regulatory roles in 286 metabolic and vascular tissue gene-protein sub-networks (“modules”). The modules and genes within were scored for CAD druggability potential. The scoring enriched for targets of cardiometabolic drugs currently in clinical use and in-depth analysis of the top-scoring modules validated established and revealed novel target tissues, biological processes, and druggable targets. This study provides an unprecedented resource of tissue-defined gene–protein interactions directly affected by genetic variance in CAD risk loci.

## Introduction

Genome-wide association studies (GWAS) have identified over 200 genome-wide significant and suggestive risk loci for coronary artery disease (CAD)^1^. Most of the CAD associated variants are in non-coding regions and likely affect disease development by regulating gene expression^2^. Several candidate genes regulated by lead risk SNPs have been suggested, predominantly by studies of expression quantitative trait loci (eQTLs)^2–4^. However, the target tissues, biological processes, and pathways through which these loci affect CAD etiology are largely unknown. For example, although it has been postulated that many loci appear to affect CAD by regulating genes in the arterial wall^1^, presumably by influencing the predominant disease process in CAD, atherosclerosis, no druggable gene targets addressing this aspect of atherosclerosis have yet been identified^2,5^. Moreover, although CAD genes by virtue of their cellular function in themselves may not be druggable, neighboring genes in the same subnetwork or pathway may very well be.

Thus in the post-GWAS era, we need to go beyond the genetic susceptibility markers identified by GWAS, and understand how these markers actually affect disease etiology^6^. This is not a trivial task, as it is unclear whether these risk loci harbor one or several disease-causing genes and whether the effects of these genes on disease are mediated in one or several tissues. Therefore, a systems genetics approach is an unbiased way not only to better understand the molecular pathophysiology of individual GWAS risk loci and candidate genes (as provided by the subnetwork analyses) but also to determine the main target tissue(s) of a risk locus.

In this study, we first used several bioinformatics strategies to prioritize candidate genes in CAD risk loci^4^. Then, we sought for immediate neighbors of genes in the affected functional gene-networks by analyzing the unique seven-tissue Stockholm Atherosclerosis Gene Expression (STAGE)^7^ datasets (GSE40231). We integrated these functional gene-networks with data on known protein–protein interactions^8^ to infer regulatory-gene and protein networks (RGPNs) in and across the seven metabolic and vascular tissues relevant to CAD. Within these RGNPs, we computed subnetworks (“modules”) using Girvan-Newman algorithm^9^ and sought those that contained at least one of the CAD candidate genes. The motivation for going beyond individual CAD candidate genes but rather to identify modules with several genes/proteins surrounding the CAD candidate gene in the networks is driven by two principal concepts. First, unlike an isolated CAD candidate gene, the module is a community of co-expressed and interacting genes and proteins and may suggest how the locus drives CAD etiology. Second, although the CAD candidate genes themselves may not be druggable, the entire module or specific neighboring genes to the CAD candidate gene may be.

Next, to assess importance and reliability for CAD, each module was scored according to the proximity of its node/gene to the CAD candidate gene(s), tissue expression patterns (CAD vs common drug toxicity tissues) of the genes, presence of a CAD mouse phenotype, and druggability. High-scoring modules were further scrutinized to assess their content of known targets for cardiovascular drugs, general drug target enrichment, and biological functions according the gene ontology (GO).

## Methods

### Gene Prioritization

Genome-wide significant (*P* < 5 × 10^−8^) and genome-wide suggestive CAD SNPs were collected from recent association studies^1,2,10,11^. The genome-wide suggestive SNPs included variants with a false-discovery rate (FDR) ≤ 5%^1^ and variants identified in the discovery analysis in “Exome array” studies^10,11^. SNPs in high linkage disequilibrium (LD) (*r*^2^ > 0.8) with each lead variant was identified with the 1000 Genomes phase 1 v3 ALL reference panel. We used a similar approach to a previous study^2^, genes were linked to CAD loci in three ways: (1) proximity to lead and high LD SNPs ascertained by searching the ENSEMBL database (GRCh38) for RefSeq annotated genes, (2) a long-range interaction between a chromosomal region containing a gene and the region containing the CAD-associated SNPs^12^, and (3) the eQTL most significantly associated with the CAD lead SNP or proxy^13–29^. A “Prioritized Gene List” was generated as described in the Results section. GWAVA software was used to identify transcribed SNPs and SNPs that alter the coding sequence^30^. Deleterious SNPs were predicted with SIFT^31^, PolyPhen^32^, and CADD^33^. Mouse phenotypes were assigned if the gene had been reported to cause an atherosclerosis phenotype^34^. All gene symbols were confirmed with the HGNC Multi-symbol checker tool.

To prioritize the most likely causal gene at each locus, we scored each of the 475 genes using six criteria for functional relations: the SNP (1) has a chromatin interaction with the gene, (2) is transcribed, (3) causes a coding change, (4) is an eQTL, and (5) is predicted to be deleterious and (6) the mouse knockout of the gene has an atherosclerosis phenotype^34^. We assigned each category a weight of *2^(L-1)*, where *L* is the rank of the category, and the score for each gene was the sum of the weighted score across the six categories (Supplementary Table 1A). The top-scoring gene at each locus was considered the most likely to be causal.

### Network Construction

To refine and further supplement the cross-tissue co-expression networks from the Stockholm Atherosclerosis Gene Expression (STAGE) study^7,35^, we added tissue-specific protein–protein interactions (PPIs) from the ConsensusPathDB (http://consensuspathdb.org) database^8^, which contains 261,085 protein interactions from 19 different resources, including IntAct^36^, HPRD^37^, and BioGRID^38^. ConsensusPathDB assigns a confidence score to each binary PPI, an aggregate score based on network-topological and annotation features (e.g., literature evidence, pathway and Gene Ontology co-annotation), with scores <0.5 denoting low confidence and those >0.95 denoting high confidence. Otherwise, the interactions were considered to be of medium confidence. We selected PPIs with confidence scores ≥0.5. To ensure the tissue-specificity of PPIs, we used gene expression data from tissues in the STAGE study^7^: atherosclerotic arterial wall (AAW), internal mammary artery (IMA), liver (LIV), skeletal muscle (SM), subcutaneous fat (SF), and visceral fat (VF). For each cross-tissue co-expression network gene, we added its protein interaction partners to the network only if both interactors were expressed in the same tissue. A gene was considered to be expressed in a particular tissue if its median expression signal was above the background level (i.e., Robust Multi-array Average (RMA) log2 intensity values ≥5.0) across all samples. Subsequently, to increase network connectivity, we also searched for PPIs within these added nodes (i.e., the first neighbors of each cross-tissue co-expression network gene), again requiring that both interactors be expressed in the same tissue.

Informed consent was obtained from all participants in the STAGE study and all the methods were approved by the Ethics Committee of the Karolinska University Hospital (Ethical approval Dnr 02-004) and performed in accordance with relevant guidelines and regulations^7^.

### Module Identification and Scoring

Gene modules within networks were identified with the Girvan-Newman algorithm^9^, implemented in R *igraph* (http://igraph.org)^39^. Modules were defined as subsets of vertices within which vertex–vertex connections were dense but between which such connections were less dense. The Girvan-Newman algorithm detects modules hierarchically by progressively removing edges from the original network. Hence, the algorithm focuses on edges that are least central and thus most likely to be “between” modules (for details, see^9^).

The scoring of the extracted modules was performed in R (R version 3.0.2, Bioconductor version 2.13)^40,41^, unless otherwise specified. BioMart^42,43^ was used to ensure the gene names matched HGNC nomenclature. Gene-wise scoring was done individually for each selected network containing modules of interest. The integrated score was the sum of the scores in the following six categories:

1. The distance to a CAD candidate gene. The shortest path of each network gene to a CAD candidate gene (including both the genome-wide significant and suggestive loci associated genes) was calculated with the *shortest.paths* function in the R/Bioconductor package *igraph*. The CAD candidate genes used are listed in Supplementary Table 1B. If a gene was a CAD candidate gene (i.e., the distance was zero), it was assigned a score of 4. If the distance was one, the gene was assigned a score of 2. If the distance was two, the gene was assigned a score of 1.
2. The expression level of a gene in CAD tissues relative to that in toxicologically relevant tissues (Tox tissues), defined as those with common drug side effects. The CAD tissues were those used in the STAGE study (liver, skeletal muscle, visceral fat, subcutaneous fat, whole blood, atherosclerotic arterial wall, and unaffected arterial wall); RMA condensed Affymetrix gene expression values were used (provided by CGN). The Tox tissues were liver, heart, kidney, cerebral cortex, pancreas, and testis; the FPKM (fragments per kilobase per million) gene-level condensed RNA-seq was downloaded from the EBI-EMBL Expression Atlas (http://www.ebi.ac.uk/gxa/experiments/E-MTAB-1733). Since liver is both a CAD tissue and a Tox tissue, it was not assigned to either category but was used instead for normalization to facilitate the comparison of Affymetrix data and RNA-seq data.
  For STAGE Affymetrix data, the RMA values per tissues were averaged (median) and normalized to expression in the reference tissue (liver) by taking the ratio. Data from atherosclerotic arterial wall and unaffected arterial wall were merged by taking the average. The average (mean) expression value for all CAD tissues was calculated from the normalized tissue expression values.
  For RNA-seq data, the zero values were replaced by an arbitrary minimum FPKM value of 0.5 (to avoid data loss upon log transformation), and genes containing “LOWDATA” or “FAIL” tags were removed. For non-unique genes, average (mean) FPKM values were calculated. The tissue expression values were normalized by calculating the ratio to expression in the reference tissue (liver). The average (mean) expression value for Tox tissues was calculated from the normalized tissue expression values.
  To correct for different dynamic ranges, the normalized expression values for CAD and Tox tissues were transformed to an interval [0,1]. For genes present in both data sets, the ratio of CAD expression to Tox expression was calculated. The scores were scaled between −2 and 2 to yield the desired weight for the summary score.
3. Vascular expression. All STAGE Affymetrix CEL files from the NCBI Gene Expression Omnibus (GEO) (series GSE40231) were loaded into Genedata Analyst software (version 8.2.4a) and MAS5 condensed. Samples from internal mammary artery and atherosclerotic aortic wall were selected. A gene was considered to be expressed in a particular vascular tissue if one or more of its probes was “present” as judged from MAS5 absent/present calls in ≥50% of samples. If a gene was present, it was assigned a score of 1.
4. Kinases and G-protein-coupled receptors (GPCRs). A list of human and mouse protein kinase coding genes from the Uniprot (http://www.uniprot.org/docs/pkinfam.txt) database was downloaded (on 9.5.2014, release 2014_04). A list of GPCR coding genes from the IUPHAR/BPS Guide to Pharmacology (http://www.guidetopharmacology.org/GRAC/GPCRListForward?class=A) was downloaded on 8.5.2014 (file targets_and_families.csv). All gene names were checked with HGNC Multi-symbol Checker (http://www.genenames.org/cgi-bin/symbol_checker). The lists of kinases and GPCRs were combined and collapsed to include only unique gene names (including HGNC and non-HGNC converted names). A score of 2 was assigned to each kinase and GPCR coding gene.
5. Existing drugs targeting the gene product. The genes measured in the STAGE study and the proteins/genes they interact with (from the ConsensusPathDB) were checked with the HGNC Multi-symbol Checker. All unique gene names (including HGNC and non-HGNC converted names) were listed and checked for drug interactions in DGIdb v2.22 (Interaction.tsv file). Genes with at least one drug in the database were assigned a score of 2.
6. Mouse phenotype. A list of all the genes that had atherosclerosis phenotypes in mice was obtained from the University of Utrecht^34^. The gene names were converted to official gene symbols with HGNC Multi-symbol Checker, and a list of all the unique gene names (including HGNC and non-HGNC converted names) was compiled. Genes with atherosclerosis mouse phenotypes were assigned a score of 1.

To score the resulting network modules, we selected those containing at least one GWAS hit and summed the integrated scores of all genes in the module. The module score was then normalized to the number of genes in the module:

$${Module}\_{Score}=\frac{\sum Integrated\_Gene\_Score}{Module\_Size}$$

### Functional Enrichment Analysis

To analyze the functional enrichment of network modules, we downloaded Gene Ontology (GO) (http://www.geneontology.org)^44^ annotations from the AmiGO 2 browser (http://amigo.geneontology.org/amigo). The GO project provides information about gene product function by using ontologies to represent biological knowledge in three classes: (1) molecular functions, (2) the biological processes they contribute to, and (3) the cellular locations where they occur (cellular components). Fisher’s exact test^45^ in R (http://www.r-project.org) was used to calculate the statistical significance of the overlaps; thereafter, the Benjamini-Hochberg (BH) procedure^46^ was used to control for the FDR.

### Network Visualization

Networks were visualized with the yFiles organic layout algorithm in Cytoscape v3.2.1^47^.

### Drug Enrichment Analysis

To test for drug target enrichment, we identified all genes targeted by drugs in the DGIdb^48^. For each module, the enrichment was calculated by using Fisher’s exact test to compare the number of genes in the module targeted by drugs to the total number of genes targeted by the drugs. We used two approaches. First, we did an enrichment analysis, with Fisher’s exact test, for general drug targets and known cardiometabolic drug targets (Supplementary Table 5). In the analysis where the enrichment of cardiometabolic drug targets was compared to other drug targets, the gene was considered cardiometabolic drug target if one or more cardiometabolic drugs targeted the gene, and other drug target if no cardiometabolic drugs targeted the gene and the gene was on the list of all genes targeted by drugs derived from the DGIdb^48^. Second, we created a dictionary of Anatomical Therapeutic Chemical Classification System (ATC) codes based on information from MedNet INN^49^ and ATC/DDD^50^. ATC codes reflect standard practices in drug administration, according to diseases for which each drug is most prescribed. Using these ATC codes, we created “drug sets”, a concept similar to gene sets but applied to drugs prescribed for the same human system. Fisher’s exact test was used to test enrichment in specific systems.

## Results

### Prioritizing CAD Candidate Genes in Risk Loci Identified by GWAS

We used integrative bioinformatics to link possible causal genes to CAD-associated SNPs^2^. In brief, we first collected 264 genome-wide significant and suggestive CAD SNPs from the most recently reported GWAS^1,10,11^. These variants corresponded to 213 independent association signals based on LD of *r*^2^ < 0.2. We then identified all proxy SNPs in high LD (*r*^2^ > 0.8) with the lead variants. Next, we linked candidate genes to lead SNPs and high LD proxies based on proximity, association with gene expression^13–29^ and long-range interaction with *trans*-genes outside the locus^12^. This approach linked 475 genes to the CAD variants (Supplementary Table 1A).

We then scored each of the genes based on functional evidence and prioritized the most likely causal gene at each locus (Supplementary Table 1A) (for more details see Methods). In total, we prioritized 184 unique CAD candidate genes identified in this fashion, of which 68 were at genome-wide significant loci (Supplementary Table 1B). Some loci were linked to more than one equally scoring gene, and no candidate gene could be assigned to 49 SNPs, as no gene had a score above zero or were excluded as the linked gene did not have an HGNC gene name.

### Identifying and Scoring Subnetworks (Modules) Containing CAD Candidate Genes

**Figure 1:**
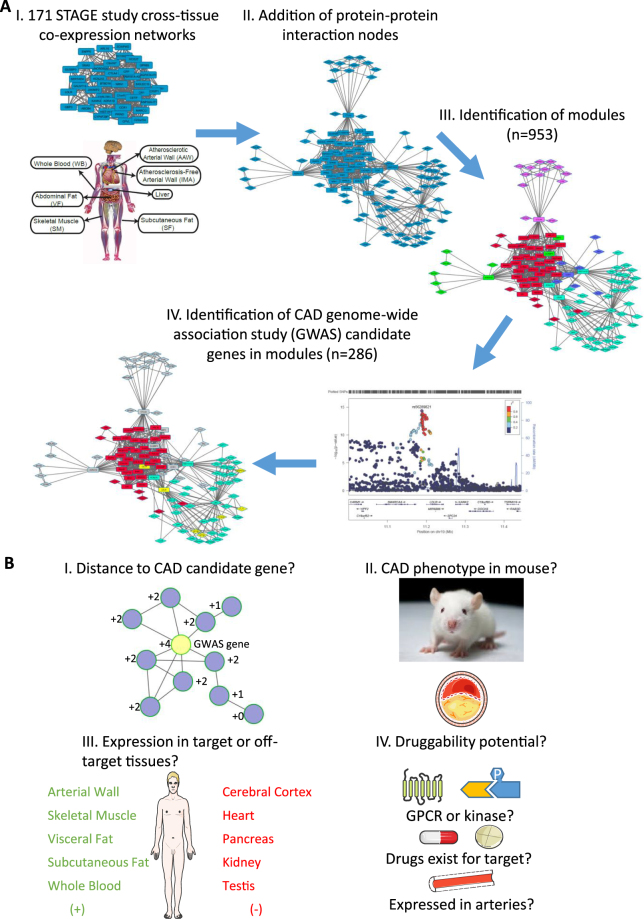
Principal analysis steps to identify and score gene/protein subnetwork modules containing CAD candidate genes. (**A**) Analysis steps to identify subnetwork modules with CAD candidate genes. (I) In step 1, 171 tissue-specific and cross-tissue co-expression networks inferred from the Stockholm Atherosclerosis Gene Expression (STAGE) study^7,35^. (II) In step II to account also for gene-protein and protein-protein interactions (PPIs), the ConsensusPathDB^8^ was used to add protein nodes to the STAGE gene networks conserving tissue integrity. (III) In step III, to extract smaller, likely functional, units Girvan-Newman algorithm was used to identify gene/protein modules within each networks resulting in 953 modules. (IV) In step IV, 286 modules affected by genome-wide significant loci (p < 5 × 10^−8^) were selected (Supplementary Table 1b). Squares indicate genes nodes from STAGE data. Diamonds represent protein nodes from ConsensusPathDB database. Color-coding highlight different modules. Yellow nodes are CAD candidate genes (*LDLR*, *CETP*, *APOB* and *PCSK9* (p < 5 × 10^−8^), *APOE* and *MAPK14* (FDR ≤ 5%)). (**B)**, Principles for scoring gene/protein subnetwork modules with CAD candidate genes. The scoring theme was set to prioritize modules in relevant tissues and biological processes harboring druggable targets against CAD. Specifically, individual nodes were scored according to (I) distance to CAD candidate gene in module, (II) genetic modification of the mouse ortholog displaying an atherosclerotic phenotype (III) expression in CAD relevant tissues (green, positive score) or in tissues commonly displaying drug toxicity (red, negative score), and (IV) the CAD druggability potential of the gene. The final CAD-feasibility score for each module was calculated from the sum of individual gene/protein node scores divided by the total number of nodes/module. Several figures in panels II-IV have been obtained and adapted from Servier Medical Art (www.servier.com) which are distributed under Creative Commons license (https://creativecommons.org/licenses/by/3.0/).

To investigate the molecular mechanisms, pathways, and regulatory networks in which the prioritized CAD genes operate within and across CAD-relevant tissues to affect CAD risk, we identified tissue-specific subnetworks containing CAD candidate genes as shown in Fig. 1A. In this process, first, we inferred regulatory gene networks (RGNs) from the 171 multi-tissue gene co-expression networks from the STAGE study^7,35^. Second, tissue-specific nodes of the RGNs were extended with published, high-confidence protein-protein interactions (PPIs) from the ConsensusPathDB database^8^ to create regulatory gene protein networks (RGPNs). The CAD relevance, as assessed by enrichment of CAD candidate genes, of adding PPIs to STAGE gene-networks was confirmed by comparing modules inferred from both STAGE data and PPIs, to modules inferred from PPIs alone (i.e. building modules from PPIs alone, without using STAGE coexpression networks as prior). The details and results from the comparison are described in the Supplementary Material (Page 1). Third, subnetworks (modules) were computed from each RGPN^51^, resulting in 953 modules whereof 449 were found to contain at least one CAD candidate gene (Supplementary Table 2). However for the subsequent analysis, we focused on the 286 modules containing at least one of the 68 prioritized CAD genes linked to a genome-wide significant risk loci (*P* < 5 × 10^−8^) (Supplementary Tables 1B and 2). Among these, the most “promising” modules from a CAD perspective were identified by calculating a CAD-feasibility score (Fig. 1B), which is an average module score based on individual scores of each of the module genes according to four criteria: (1) proximity to the module CAD candidate gene(s), (2) genetically modified in mice with an atherosclerosis phenotype, (3) target tissue (i.e., toxic or CAD-relevant), and (4) druggability (e.g., GPCRs and kinases as the two largest druggable gene families in human genome^52^). Based on the weights assigned to each scoring category (see Methods), the score can range from −2 to 12. The 25 top-scoring modules are shown in Table 1.

**Table 1:** Key features of the top 25 modules.

Module ID	Size	CAD-feasibility score	Main tissue	CAD candidate genes	Main GO biological process term	Drug target nodes (*n*)	Drug target enrichment (*P* value)	Cardiometabolic drug targeted nodes	Cardiometabolic target enrichment (*P* value)
119_6	10	5.71	VF	LDLR*	Chemotaxis	9	3.31E-08	2	4.25E-03
18_4	6	5.65	VF	COL4A1	Platelet degranulation	5	1.12E-04	1	5.83E-02
130_2	65	5.43	SM	IGF1R, SHC1, IL6R	Epidermal growth factor receptor signaling pathway	45	2.16E-27	0	1.00E + 00
36_4	9	5.37	SM	ADM	Vein smooth muscle contraction	6	1.46E-04	1	8.62E-02
142_4	14	5.35	AAW	LRP1, *FN1	Complement activation	11	1.29E-08	4	9.33E-06
139_5	4	5.16	LIV	SLC22A5, SLC22A3	Quaternary ammonium group transport	3	5.64E-03	0	1.00E + 00
1_5	5	5.09	IMA, AAW, WB	ADM	Complement activation, alternative pathway	3	1.29E-02	1	4.88E-02
134_1	38	4.98	IMA, SM	RELA, TNF, *SHC1, LRP1*	Innate immune response	22	8.60E-12	4	5.67E-04
17_3	12	4.93	IMA, WB, VF	LRP1*	Extracellular matrix disassembly	8	1.02E-05	4	4.69E-06
82_4	37	4.93	AAW, SF	LRP1, *FN1, COL4A1	Extracellular matrix organization	25	1.78E-15	6	1.85E-06
171_2	59	4.85	SF	SHC1, MRAS	epidermal growth factor receptor signaling pathway	41	3.39E-25	0	1.00E + 00
108_3	75	4.83	VF	PLCG1, LRP1, *ITGB5, SHC1	Blood coagulation	43	2.04E-21	4	6.95E-03
163_1	46	4.78	SF	TNF, *FN1	Extracellular matrix organization	32	5.17E-20	4	1.18E-03
137_2	111	4.76	SM	PLCG1, BCAR1, SHC1, FLT1	Peptidyl-tyrosine phosphorylation	61	2.72E-28	3	1.00E-01
126_2	44	4.74	SM	LRP1, *FN1, COL4A1	Extracellular matrix organization	28	3.89E-16	6	5.27E-06
89_3	58	4.73	SM	FN1, COL4A1	Extracellular matrix organization	34	1.13E-17	8	1.32E-07
143_5	56	4.72	IMA	RELA, NOS3, *IGF1R, SMAD3	Innate immune response	36	1.51E-20	2	1.08E-01
91_4	29	4.70	IMA; SF, VF, SM	LDLR, *APOE, *SCARB1, NOS3*	Receptor-mediated endocytosis	17	1.55E-09	3	3.01E-03
116_1	10	4.68	LIV, SF	FURIN	Caveolin-mediated endocytosis	8	1.16E-06	1	9.53E-02
84_3	63	4.65	VF	FN1, COL4A1	Blood coagulation	50	4.06E-35	19	7.30E-23
10_3	15	4.57	AAW, LIV, SF,VF	PLG*	Extracellular matrix disassembly	10	7.37E-07	4	1.26E-05
72_6	7	4.56	IMA	EDNRA*	Cell division	3	3.77E-02	1	6.77E-02
130_3	108	4.56	SM	MAPK14, RELA, TNF, *FN1	Positive regulation of NF-kappaB transcription factor activity	58	2.76E-26	3	9.44E-02
69_5	31	4.54	AAW	FN1: COL4A2: COL4A1	Extracellular matrix organization	16	5.53E-08	1	2.67E-01
124_1	43	4.52	SM	MAPK14: RELA: SMAD3	Activation of MAPKK activity	23	2.68E-11	1	3.50E-01

The table is sorted by module CAD-feasibility score and shows the tissue(s) of the module, CAD candidate genes in the module with genes targeted by cardiometabolic drugs marked with asterisk, the most highly enriched Gene Ontology biological processes (GOBP) category, and number of genes in the module that are targeted by cardiometabolic drugs and by all known drugs. *P* value of the enrichments is based on Fisher’s exact test.

### Known Cardiometabolic Drug Targets Validate Module Scoring Theme

**Figure 2:**
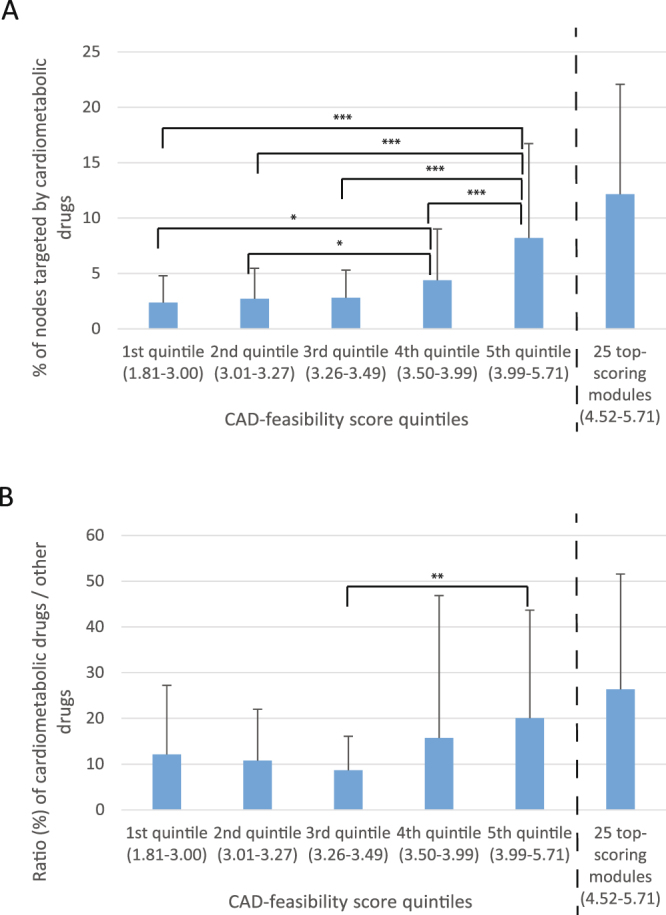
Correlation between CAD-feasibility score and cardiometabolic drug target gene/protein enrichment. (**A**) The plot shows the 286 modules divided in to five equal size (57–58 modules in each) groups based on the CAD-feasibility score and the 25 top-scoring modules (which is a sub-group of 5^th^ quintile). For each module group the arithmetic mean of the % of nodes targeted by cardiometabolic drugs in each module is shown together with the standard deviation. The score range for the each quintile is shown below the bars. The statistical difference between the quintile groups were tested with Kolmogorov-Smirnov two-group test; the statistically significant comparison are indicated with the arches above the bars: *p < 0.05, **p < 0.01, ***p < 0.001. (**B**) The plot shows the 286 modules divided in to equal size quintiles (57–58 modules in each) groups based on the CAD-feasibility score, and the 25 top-scoring modules (which is a sub-group of 5^th^ quintile). For each module group the arithmetic mean of the the ratio of cardiometabolic drugs targets versus all other drugs targets is shown together with the standard deviation. The statistical difference between the quintile groups were tested with Kolmogorov-Smirnov two-group test; the statistically significant comparison are indicated with the arches above the bars: **p < 0.01.

Although we judge the categories for scoring the modules as adequate and reasonable, the scoring categories and weights are arbitrary by nature. We therefore sought means to assess the validity of the module-scoring theme by taking advantage of known target genes of drugs currently in clinical use for CAD and CAD-related risk factors retrieved from the websites of the U.S. Food and Drug Administration (fda.gov) and the open drug resource drugs.com. Reassuringly, many of the 286 modules were enriched for cardiometabolic drugs (Supplementary Table 2 and Supplementary Text page 2) and modules strongly enriched in gene/protein targets for these cardiometabolic drugs also tended to have high CAD-feasibility scores (Supplementary Figure 1). This trend was further reflected in that the top scoring quintiles (4^th^ and 5^th^ quintile) of the modules were significantly (Kolmogorov-Smirnov test) more enriched in genes targeted by cardiometabolic drugs than lower scoring quintiles (Fig. 2A). Moreover, the 25 top-scoring modules were found even further enriched in cardiometabolic drug targets (i.e., top 9^th^ percentile of modules with >4.5 points scores, versus the remaining 33 modules in the top quintile, 12.2 ± 9.9% vs. 5.2 ± 5.8%, *P* = 0.0016 Kolmogorov-Smirnov test, Table 1). The fact that high scoring modules were also often enriched for targets of non-cardiometabolic drugs (Table 1 and Supplementary Table 2) was not surprising since the scoring theme favors genes/proteins that are known drug targets. However the observation that the ratios of cardiometabolic to other drug targets were the highest in top scoring modules is noteworthy (Fig. 2B) as this underscores their possible relevance for understanding CAD and to identify novel targets associated with genetic risk.

### Characteristics of 25 Top-scoring Modules and Drug Target Enrichment

The 25 top-scoring gene/protein modules had four to 111 nodes (Table 1). Seven modules were active in several CAD tissues in STAGE, and 18 modules were tissue-specific. Eight of 25 modules had only one CAD candidate gene, six had two respective genes, five had three, and six had as many as four (Table 1). Thus, although we identified an average of ~1.1 candidate genes per CAD risk locus (184 genes in 169 risk loci), many candidate genes/proteins were found in the same module, implying cumulative effects across independent loci^3^. Among the 25 top-scoring modules, 22 had an average of four genes/proteins targeted by cardiometabolic drugs; in 13 modules, the genes/proteins targeted by cardiometabolic drugs coincided with the CAD candidate gene (*P* < 0.05, Fisher’s exact test; Table 1 and Supplementary Table 5). Importantly, however, GO and pathway analyses (Supplementary Table 7) showed that the functional enrichments of these 22 modules mostly did not coincide with the pathway or tissue targeted by the drugs. Thus, cardiometabolic drugs in clinical use today may have unknown pleiotropic effects outside their assumed target tissues and pathways.

### Drug Targets and Categories in the 25 Top-scoring Modules

We also tested for the distribution of drug categories that targeted any of the genes in the 25 top-scoring modules (Table 2). To this end, we used the ATC (Anatomic Therapeutic Chemical classification system) codes from MedNet INN^49^ and ATC/DDD^50^. As expected, we noted a significant enrichment (Fisher’s exact test right-tail *P* < 0.05) in cardiovascular drugs targeting the genes/proteins in four of the 25 top-scoring modules, and two modules were enriched in drugs targeting blood and blood-forming organs), with one overlap (module 84_3). However, the most often enriched ATC categories were antineoplastic and immune-modulating agents (in 13 of the 25 top modules), suggesting some of these CAD modules contain target genes/proteins for non-cardiometabolic drugs that may be repurposed for CAD.

**Table 2:** ATC groups most represented by drugs that target gene products of the top 25 modules.

ATC group	ATC group code	Significant modules	
		*n*	ID
Antineoplastic and immunomodulating agents	L	13	18_4, 130_2, 134_1, 82_4, 171_2, 108_3, 137_2, 126_2, 143_5, 91_4, 116_1, 130_3, 124_1
Cardiovascular system	C	4	36_4, 17_3, 84_3, 72_6
Musculoskeletal system	M	4	17_3, 82_4, 163_1, 69_5
Blood and blood-forming organs	B	2	84_3, 10_3
Nervous system	N	1	124_1
Genitourinary system and sex hormones	G	1	130_3
Sensory organs	S	1	163_1
Alimentary tract and metabolism	A	0	
Dermatologicals	D	0	
Systemic hormonal preparations, excluding sex hormones and insulins	H	0	
Anti-infectives for systemic use	J	0	
Antiparasitic products, insecticides, and repellents	P	0	
Respiratory system	R	0	
Various	V	0	

Listed are the ATC groups and the number and IDs of the modules significantly enriched (Fisher’s exact test, right-tail *P* < 0.05) in targets of ATC drug groups. ATC, Anatomical Therapeutic Chemical Classification System.

### Enrichment of the 25 Top-scoring Modules in Biological Processes

**Figure 3:**
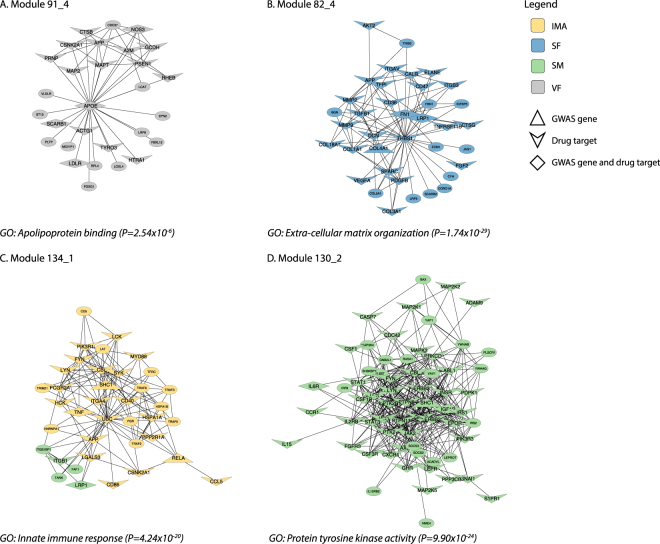
Examples of subnetwork modules. (**A**) Cholesterol and lipoprotein metabolism and homeostasis (Module 91_4); (**B**) Extracellular matrix organization and regulation, blood coagulation and platelet activation (Module 82_4). (**C**) Innate immune response (Module 134_1). (**D**) Cellular signaling (Module 130_2). In the figures, the GO biological process term with the lowest *P* value (Benjamini-Hochberg; Fisher’s exact test) is shown. IMA, internal mammary artery; SF, subcutaneous fat; SM, skeletal muscle; VF, visceral fat.

To help establish the function, pathway, and druggability of CAD candidate genes, we annotated the 25 top-scoring modules by GO biological process (Table 1, Supplementary Table 7). All modules were enriched (*P* < 6.12 × 10^−6^, Fisher’s exact test) in GO categories that were largely non-overlapping between the modules, suggesting that each module has a relatively distinct biological function. However, several categories were over-represented, including extracellular matrix organization and disassembly, blood coagulation, platelet activation, innate immune response, complement activation, leukocyte migration, and various cellular signaling mechanisms. In Fig. 3 and as discussed below, four of the top-scoring 25 modules were highly interesting, as judged from their tissue-belonging, CAD candidate genes, and representative biological processes. The most relevant biological products of these pathways are involved in extracellular matrix organization, blood coagulation, platelet activation, innate immune response, complement activation and leukocyte migration. Similar characteristics for all 286 module are available in the Supplementary Material (See Supplementary Information).

## Discussion

Herein, we applied comprehensive bioinformatics to a number of both public and unique datasets of CAD to create a first repository of network modules associated with inherited risk for CAD as identified by GWAS^1,10,11^. In tissue-specific manner, these networks describe both established and new biological processes and pathways providing new insights into the mechanisms by which genetic risk affects CAD etiology. Furthermore, the network repository also delivers new insights of both CAD-established, and novel drugs and drugs targets that now can be further evaluated to reassess their effects on CAD and related risk factors. In sum, the identified network modules represent an unprecedented resource for tissue-specific gene–protein interactions directly affected by genetic variance in CAD risk loci and should help expedite their translation into new opportunities for CAD diagnostics and therapies.

Module 91_4 was highly enriched in GO categories related to lipoprotein and cholesterol metabolism (Fig. 3A, Supplementary Table 7) and was mainly active in visceral fat (Fig. 3A and Table 1). Its four CAD risk candidate genes (*LDLR*, *APOE*, *SCARB1*, and *NOS3*) (Fig. 3A and Supplementary Tables 1 and 8) are well-established in lipoprotein and nitric oxide/cGMP metabolism and have been identified as candidate genes by GWAS^1,53,54^. The cardiometabolic drugs targeting *LDLR* and *APOE (*e.g. statins), are among the most widely used cardiometabolic therapies worldwide^55^, and *NOS3* genotypes are indirectly related to activators of soluble guanylyl cyclase (e.g. riociguat) or inhibitors of phosphodiesterase 5 A (e.g. sildenafil) (Table 1 and Supplementary Tables 5 and 8)^56^. Thus, this module serves as a proof-of-concept for our analysis pipeline. This module also harbored genes/proteins that are known targets of antineoplastic and immunomodulating agents (five drugs, Fisher’s exact test right-tail *P* = 0.006; Table 2 and Supplementary Table 9). Several other module genes, including immediate neighbors of CAD candidate genes (e.g., *CSNK2A1* and *HTRA1*) are also druggable (Fig. 3A and Supplementary Table 8) and thus may be repurposed for modulating the effects of this module in the context of atherosclerosis prevention.

Ten of the top 25 modules were found to be implicated in extracellular matrix organization and disassembly, blood coagulation, or platelet degranulation/activation (Table 1). Module 82_4, a representative example (Fig. 3B, Supplementary Table 7), has three CAD candidate genes: *LRP1*, *COL4A1* and *FN1* (Fig. 3B, Table 1, and Supplementary Tables 1 and 8). This module mainly contained genes expressed in subcutaneous fat (Fig. 3B and Table 1). Four first or second neighbors of the CAD candidate genes in this module are targets of current cardiometabolic drugs: *TFPI* (dalteparin), *ITGB3* (abciximab and eptifibatide), *MMP2* (captopril) and *VEGFA* (dalteparin) (Table 1, Supplementary Tables 5 and 8), and for these gene-drug pairs CAD relevant physiological effects have been reported (for more details and references see https://www.drugbank.ca)^57–63^. These genes are good examples of module genes that are directly adjacent to GWAS CAD candidate genes and that may be alternative or better drug targets than the GWAS-derived risk genes themselves. The drugbank database (https://www.drugbank.ca) also reports CAD candidate gene *LRP1* and its first neighbor *CALR* as targets for the fibrinolytic agent tenecteplase (recombinant tissue plasminogen activator tPA). These drug-gene links are based on the binding of the LRP1 and CALR proteins to tPA in *in-vitro* models^64,65^ and the physiological relevance of these interactions have not been established (for further discussion on the limitations of drug-gene interaction databases see below). Moreover, this module contains many nodes that (1) are druggable (the CAD candidate genes *FN1* and *MMP9*^66^ and their neighbors such as *THBS1*), (2) have an atherosclerosis phenotype in mice, and (3) have a preferable non-toxic target tissue (Fig. 3B and Supplementary Table 8). This module also harbored nodes targeted by antineoplastic and immunomodulating agents (n = 7, Fisher’s exact test right-tail *P* = 0.001) and by drugs targeting the musculoskeletal system (n = 4, Fisher’s exact test right-tail *P* = 0.001) (Table 2 and Supplementary Table 9).

Module 134_1 is representative of four modules enriched in biological processes related to the innate immune response (Fig. 3C, Table 1, and Supplementary Table 7). It is a cross-tissue module involving both the arterial wall and skeletal muscle (Fig. 3C and Table 1) and contains four CAD candidate genes: *RELA*, *TNF*, *SHC1*, and *LRP1* (Table 1 and Supplementary Tables 1 and 8). Pentoxifylline regulates *TNF* production and release from white blood cells^67,68^, however the physiological and clinical relevance of this in terms of CAD has not been tested (Table 1 and Supplementary Tables 5 and 8). Several other nodes in the module (e.g., *LYN* and *SYK*) are druggable, have atherosclerosis phenotypes in mice, and have preferable target tissues (Supplementary Table 8) and thus may be interesting targets for new CAD therapies. Other well-established drugs targeting this module are antineoplastic and immunomodulating agents (n = 7, Fisher’s exact test right-tail *P* = 4.78 × 10^−12^) (Table 2 and Supplementary Table 9).

Several signaling pathways are among the top GO biological processes in the top-scoring modules (Table 1). For example, module 130_2 is enriched in pathways for epidermal growth factor receptor signaling, fibroblast growth factor receptor signaling, JAK-STAT cascade involved in growth hormone signaling, neurotrophin TRK receptor signaling, Fc-epsilon receptor signaling, cytokine-mediated signaling, and insulin receptor signaling (Fig. 3D and Supplementary Table 8). This module contains three CAD candidate genes (*IGF1R*, *SHC1*, and *IL6R*) and is specific to skeletal muscle (Table 1 and Fig. 3D). Unlike the preceding examples, this module is not targeted by current cardiometabolic drugs (Table 1). However, it contains several CAD candidate genes (e.g., *IGFR1*) and their immediate neighbors (e.g., *CXCR4*) that are druggable, have atherosclerosis phenotypes in mice, and act in a non-toxic tissue (Supplementary Table 8). The drugs targeting this module are mainly used as antineoplastic and immunomodulating agents (54 drugs, Fisher’s exact test right-tail *P* = 2.32 × 10^−58^) (Table 2 and Supplementary Table 9).

As further validation and extension of our approach and findings, we had a more detailed look at the atherosclerosis related mouse phenotypes reported for the CAD risk candidate genes and their neighbors in the top modules^34,69^. In module 91_4, all the four CAD risk candidate genes (*LDLR*, *APOE*, *SCARB1*, and *NOS3*) display protective role against atherosclerosis (i.e. gene deletion increases atherosclerosis risk/phenotype) by regulating cholesterol or blood pressure levels^70–73^. Interestingly, several of their neighboring genes (*LCAT*, *VLDLR*, *PLTP*, *APP*) in the modules have driver roles for atherosclerosis (i.e. gene deletion decreases atherosclerosis risk/phenotype)^74–77^ and thereby could be attractive targets for drug inhibition. Mouse models of several CAD risk candidate genes (e.g. *SCH1*, *RELA* and *FN1*) in modules 82_4, 130_2 and 134_1 display phenotypes related macrophage accumulation and foam cell formation in atherosclerotic lesions^78–80^, thereby highlighting the roles of different immune system elements and signaling pathways these modules regulate in CAD. The mouse phenotypes also confirm that the modules contain attractive CAD drug target candidates, as many of the genes have a driver role for atherosclerosis (e.g. *TNF* and *FN1*) (for more details and references see^69^).

The multifactorial etiology of CAD and the diversity of causative mechanisms involved makes it likely that multiple pathways have to be addressed by medical treatment in order to neutralize risk. In this sense, the resource presented here can provide new ideas for combination therapies for the treatment of CAD. Combination therapies could provide beneficial therapeutic effects either by acting within a single module or across several modules, depending to which extend the relevant mechanism is being affected. Within module co-treatment of two different node targets with two (or more) medicines could result in a more efficient reduction of a single CAD driving mechanism and cause. A potential example of this is co-treatment with statins and PCSK9 inhibitors to regulate cholesterol levels and atherosclerosis^81^, as *PCSK9* was identified in the same module with statin targets *LDLR* and *APOE* (see module 112_1 in Supplementary Table 2 and Supplementary Material). Cross module co-treatment could potentially lead to reduction of more than one of the CAD driving mechanisms and causes (e.g. regulation of cholesterol levels and platelet functions) which could lead to efficient overall improvements in CAD patients. A potential example for this would be co-targeting of modules 91_4 and 82_4 with statins and anticoagulants, which could have beneficial effect for treating atherosclerosis and indeed has been shown to be beneficial in the recent COMPASS trial^82^. It is also feasible that modules like we have discovered in our study will help to find new potential candidates for drug repositioning in CAD.

In our approach, we prioritized genes coding for kinases and GPCRs as they represent the largest druggable gene families in the human genome. Hence the reported top modules were found to contain several kinases and GPCRs, both for which (1) drug compounds already exists (e.g. *CXCR4* and *CCR1* in module 130_2) in different stages of drug life cycle (development to market) and for which (2) current drug compounds are yet to be reported (e.g. *TRIB3* in module 82_4). The first category provides potential opportunities for drug compound repurposing^25^ and second one for new drug development in CAD.

We acknowledge several possible limitations to our study. First, for our prioritization of CAD genes there are limitations inherent by the available data. For example, some effects of disease-associated variants on gene expression will be missed as we did not have data from all CAD-relevant cell types or tissues. This is likely reflected in our results in that we were unable to link genes to every CAD locus. Also, the information we used from mouse studies is limited because not all mouse genes have been studied for CAD related phenotypes, and are further potentially biased since negative results are often not reported. We also chose to exclude the results we found from other animal models of atherosclerosis from the analysis, since these studies are sparse and often inspired by preceding mouse experiments. Thus, their inclusion would potentially further bias our results. Second, the assessments of the tissue specificity and cross-tissue signaling in the combined gene expression and PPI networks may have limitations as the PPI data did not derive from specific tissues nor from CAD individuals as does the STAGE data^7^. However, as outlined in the Supplementary Materials (Page 1), extending PPIs to existing nodes in the STAGE gene networks was essential to infer modules relevant to CAD. We also used other databases in addition to STAGE and PPIs with their own limitations such as lacking filtering mechanisms for excluding false positives and negatives. Furthermore, many of these datasets are under continuous development and refinement. Thus results presented here will be subjected to changes when rerun as the data sources then likely will have been updated. Finally, the reported links between pathways and drugs are often based on indirect or pleiotropic effects of questionable quantitative relevance. However, these limitations do not, in our view, invalidate any of the reported results or used methodology but merely point to the fact that they can be further refined in future re-evaluations.

In conclusion, we present a unique strategy, that is new and innovative, and represents a significant improvement and as such, a substantial contribution toward a more integrative understanding of the molecular processes driving CAD and its therapeutic modulation.

## Electronic supplementary material

Supplementary material

Supplementary Table 1

Supplementary Table 2

Supplementary Table 3

Supplementary Table 4

Supplementary Table 5

Supplementary Table 6

Supplementary Table 7

Supplementary Table 8

Supplementary Table 9

Online Supplementary Material
