# Supplementary material for: Network analysis of coronary artery disease risk genes elucidates disease mechanisms and druggable targets
Source: Sci Rep. 2018 Feb 21;8:3434. doi: 10.1038/s41598-018-20721-6 (PMC5821758; doi:10.1038/s41598-018-20721-6)
Supplement: Supplementary file 1 — Supplementary material [file 41598_2018_20721_MOESM1_ESM.doc]

**SUPPLEMENTARY MATERIAL**

**Network analysis of coronary artery disease risk genes elucidates disease mechanisms and druggable targets**

Harri Lempiäinen^1^, PhD; Ingrid Brænne^2,#^, PhD; Tom Michoel^3,4,#^, PhD; Vinicius Tragante^5,#^, PhD; Baiba Vilne^6,7,#^, PhD; Tom R. Webb^8,#^, PhD; Theodosios Kyriakou^9^, PhD; Johannes Eichner^1^, PhD; Lingyao Zeng^6^, PhD; Christina Willenborg^2^, PhD; Oscar Franzen^10^, PhD; Arno Ruusalepp^4^, PhD; Anuj Goel^9^, MSc; Sander W. van der Laan^11^, PhD; Claudia Biegert^1^, MSc; Stephen Hamby^8^, PhD; Husain Talukdar^12^, PhD; Hassan Faroughi Asl^12^, PhD; CVgenes@Target Consortium; Gerard Pasterkamp^11,13^, MD, PhD; Hugh Watkins^9^, MD, PhD; Nilesh J. Samani^8^, MD; Timo Wittenberger^1^, PhD; Jeanette Erdmann^2^, PhD; Heribert Schunkert^6,7^, MD; Folkert W. Asselbergs^5,14^, MD, PhD; Johan L. M. Björkegren^4,10,12^*, MD, PhD

**CAD Relevance of Adding PPIs to STAGE Networks**

The relevance of STAGE RGNs^1^ to CAD etiology was recently reported.^2^ It is likely that adding known PPIs to the STAGE networks (as we did here before the module analysis) increases the relevance of the resulting RGPNs to CAD. However, the extent to which it does so is unknown. Moreover, many of the resulting 953 modules had few or even no gene nodes from the STAGE RGNs but consisted at times solely of PPI nodes (Supplementary Table 3). To address this issue, we compared the 953 modules inferred from both STAGE data and PPIs to 528 modules inferred from PPIs alone.

In examining the node overlap between these two sets of modules (n=503,184 comparisons), only five modules had 80% or higher overlap (Supplementary Table S4). Thus, the 953 STAGE-PPI modules could not be rediscovered by using PPI alone. Moreover and importantly in terms of CAD relevance, the relative content of CAD candidate genes was significantly higher in the 953 STAGE+PPI modules than in the 528 PPI-only modules (16.5% (n=158/953) vs. 2.6% (n*=*14/528)). Accordingly, using RGNs from the multi-tissue CAD-resource STAGE as priors before adding PPIs appeared to be highly relevant and important for the ensuing node composition of the modules, even though some of these modules consisted solely of PPIs.

**Module Enrichment of Known Cardiometabolic Drug Targets**

Many of the 286 prioritized CAD candidate gene containing modules were highly enriched in the cardiometabolic drug targets (Supplementary Table 2). The average ratio of cardiometabolic drugs targets versus all other drugs targets for the 286 modules was 13.5%. The list of cardiometabolic drugs that we used contains 126 drug (Supplementary table 5), whereas DGIdb database contains over 9’000 drugs^3^, and thereby by random gene/protein selection one would expect to find less that 1.5% of drugs targeting the nodes to be in cardiometabolic treatment use. This illustrated that by using the CAD patient derived STAGE networks as starting point and selecting the modules that contain CAD candidate genes we have already selected and enriched for modules with high CAD treatment potential.

**Supplementary figures**


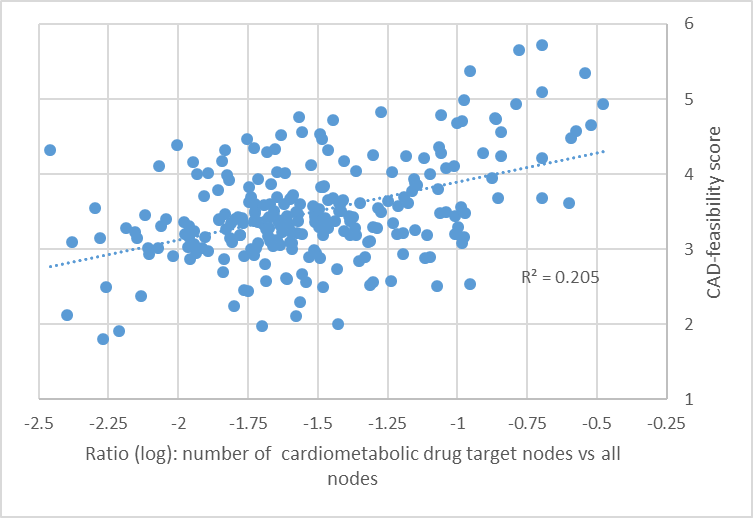


**Supplementary Figure 1. Correlation of CAD-feasibility score and cardiometabolic drug target enrichment.** The plot is shown for the modules containing at least one of the 68 CAD candidate genes identified in genome-wide significant risk locus and containing at least one node targeted by a cardiometabolic drug (n=249). The CAD-feasibility score is plotted on the *y*-axis. The ratio of cardiometabolic drug target nodes to all nodes in a module is plotted on the *x*-axis. The cardiometabolic drug target enrichment (ratio) was log transformed to correct for the skew of the nontransformed data; the skew after log transformation is 0.32. The Pearson *r* is 0.4528 and *r*^2^ is 0.205.

**SUPPLEMENTARY TABLE LEGENDS**

**Supplementary Table 1. Prioritization of CAD candidate genes to GWAS loci (A)** Linking and scoring the 264 GWAS loci to genes. The 6 scoring categories and the total score for the SNP-to-Gene link are shown. **(B)** 184 genes based on selecting the top scoring gene per each genome-wide significant (P<5x10^-8^) and suggestive (FDR 5%) locus. Loci where no genes gained a score greater than zero are excluded from the list.

**Supplementary Table 2. Characteristics of all 953 subnetwork modules.** The module name, CAD-feasibility score (only for modules containing genome wide significant (p<5x10^-8^) CAD candidate genes), size, GWA genes and results from Gene Ontology biological processes and drug enrichment analysis are shown.

**Supplementary Table 3.** **Characteristics of subnetwork modules containing CAD candidate genes.** For each module, the number of nodes originating from the STAGE cross-tissue co-expression networks (3^rd^ column), added by cataloguing the protein-protein interaction (PPI) data from ConsensusPathDB (4^th^ column) and their ratio (5^th^ column) are listed. Module GWAS hits (6^th^ (P<5x10^-8^) and 7^th^ column (FDR 5 %)) and their origin (i.e., STAGE or PPIs in the 8^th^ and 9^th^ column, respectively).

**Supplementary Table 4.** A one-by-one comparison between the module originating from the STAGE cross-tissue co-expression networks supplemented with protein-protein interactions (PPIs) from ConsensusPathDB (1^st^ column) and modules originating solely from protein-protein interaction (PPI) data (2^nd^ column). The number of nodes in the respective modules (3^rd^ and 4^th^ column, respectively), as well as the number and listing of common nodes in both modules (5^th^ and 6^th^ column, respectively) is given. Bi-directional overlap ratios (7^th^ and 8^th^ column, respectively) were calculated to determine module “similarity”.

**Supplementary Table 5. List of drugs currently used for CAD treatment in the clinics.**

**Supplementary table 6. Results from the comparison of the drug class enrichment of the modules that contain a genome-wide significant (p<5x10^-8^) loci associated CAD candidate genes (n=286, sheet 1 top panel) to the modules that do not contain genome-wide significant loci associated CAD candidate genes (n=667, sheet 2).** Results from overrepresentation analysis (Fisher’s Exact test) is shown on the bottom panel of sheet 1.

**Supplementary Table 7.** **Gene Ontology enrichment analysis results**. Molecular functions, biological processes and cellular component enrichment analysis results for the top 25 modules are shown.

**Supplementary Table 8.** **Gene level scoring and drugging analysis results**. Gene level scoring and drugging details for the top 25 modules are shown (one sheet per module).

**Supplementary Table 9.** **Drug enrichment analysis of the modules**. The ATC drug code enrichment analysis results for the top 25 modules are shown (one sheet per module).

**ONLINE SUPPLEMENTARY MATERIAL**

The online supplementary material table provides information of all the 953 modules reported in the manuscript and includes information of all the nodes and edges in the modules, together with the node characteristics (information of the gene tissue, druggability and GWAS status (gwasdrug(where 1 = CAD candidate gene, 2 = drug target, 3 = CAD candidate gene AND drug target, 0 = none of the above)), and top 10 GO categories for each module.

**SUPPLEMENTARY REFERENCES**

1. Hääg *et al.* Multi-organ expression profiling uncovers a gene module in coronary artery disease involving transendothelial migration of leukocytes and LIM domain binding 2: The Stockholm Atherosclerosis Gene Expression (STAGE) study. *PLoS Genet.* **5,** (2009).

2. Talukdar, H. A. *et al.* Cross-Tissue Regulatory Gene Networks in Coronary Artery Disease. *Cell Syst.* **2,** 196–208 (2016).

3. Wagner, A. H. *et al.* DGIdb 2.0: Mining clinically relevant drug-gene interactions. *Nucleic Acids Res.* **44,** D1036–D1044 (2016).
